# Loss of Glucocorticoid Receptor Expression by DNA Methylation Prevents Glucocorticoid Induced Apoptosis in Human Small Cell Lung Cancer Cells

**DOI:** 10.1371/journal.pone.0024839

**Published:** 2011-10-03

**Authors:** Paul Kay, George Schlossmacher, Laura Matthews, Paula Sommer, Dave Singh, Anne White, David Ray

**Affiliations:** 1 Faculty of Medical and Human Sciences, Manchester Academic Health Sciences Centre, University of Manchester, Manchester, United Kingdom; 2 Faculty of Life Sciences, Manchester Academic Health Sciences Centre, University of Manchester, Manchester, United Kingdom; 3 School of Biological Sciences, University of KwaZulu-Natal, Durban, South Africa; Univesity of Texas Southwestern Medical Center at Dallas, United States of America

## Abstract

Human small cell lung cancer (SCLC) is highly aggressive, and quickly develops resistance to therapy. SCLC cells are typically insensitive to glucocorticoids due to impaired glucocorticoid receptor (GR) expression. This is important as we have previously shown that expression of a GR transgene induces cell death *in-vitro*, and inhibits tumor growth *in-vivo*. However, the underlying mechanism for loss of GR expression is unknown. The SCLC cell line, DMS79, has low GR expression, compared to non-SCLC cell lines and normal bronchial epithelial cells. Retroviral GR expression in DMS79 cells caused activation of the apoptotic pathway as evidenced by marked induction of caspase-3 activity. Methylation analysis of the GR promoter revealed some methylation in the 1D, and 1E promoters of the GR gene, however the ubiquitous constitutively active 1C promoter was heavily methylated. In the 1C promoter there was a highly significant increase in DNA methylation in a panel of 14 human SCLC cell lines compared to a mixed panel of GR expressing, and non-expressing cell lines, and to peripheral blood mononuclear cells. Furthermore, within the panel of SCLC cell lines there was a significant negative correlation seen between methylation of the 1C promoter, and GR protein expression. Reversal of GR gene methylation with DNA methyltransferase inhibition caused increased GR mRNA and protein expression in SCLC but not non-SCLC cells. This resulted in increased Gc sensitivity, decreased Bcl-2 expression and increased caspase-3 activity in SCLC cells. These data suggest that DNA methylation decreases GR gene expression in human SCLC cells, in a similar manner to that for conventional tumor suppressor genes.

## Introduction

Changes in the epigenetic status of genes are important in many human diseases, but particularly in cancer [1]. Some of these are mediated by changes in the expression and activity of DNA methyltransferases, DMNT1, DNMT3A and DNMT3B [2], [3]. Cancer cells characteristically have hypermethylated CpG islands associated with tumor suppressor genes [1], and DNMT1 is reported to be over-expressed in lung cancer patients who smoke [4]. Recent studies have identified that a tobacco smoke carcinogen, nitrosamine 4-(methylnitrosamino)-1-(3-pyridyl)1-butanone (also known as nicotine derived nitrosamine ketone; NNK) not only induces gene mutations, but also increases hypermethylation of multiple tumor suppressor gene promoters including cyclin dependent kinase inhibitor 2A (p16ink4a), death associated protein kinase 1 (dapk1), and retinoic acid receptor b (rarb) [4]. Smoking is the major risk factor for human small cell lung carcinoma, and smoking is itself associated with methylation of more than 20 tumor suppressor genes [5], [6].

Many factors can affect glucocorticoid sensitivity, including genetic variation at the GR gene locus, and expression of interacting proteins [7]–[10]. We have previously shown that human SCLC cell lines are resistant to glucocorticoid (Gc) hormones and drugs and that this resistance is due to impaired GR expression [11], [12]. Restoration of GR expression in the cells by transfection, or viral transduction is sufficient to restore Gc sensitivity [12]. However, more importantly, restoration of GR expression is also sufficient to induce apoptosis of SCLC cells both in-vitro [13], and also in a xenograft model [14]. This raises the possibility that GR is a novel tumor suppressor gene for SCLC, and that loss of GR expression is implicated in SCLC pathogenesis.

The GR gene (NR3C1) is a ubiquitously expressed, ligand activated transcription factor, a member of the nuclear receptor superfamily. There is a single copy gene on chromosome 5, but its structure is complex, and relatively little is known about how transcription is regulated from it [15]. Transcription is controlled by 9 promoters, each associated with an alternative transcription start site. Seven of these promoters are clustered together in a CpG island, a common feature of housekeeping genes [16]. All transcripts generate authentic full-length GR protein as the translation start site is located in the common exon 2.

The GR is activated by Gc hormones from the adrenal cortex, under tight control of the hypothalamic-pituitary-adrenal (HPA) axis. This axis is under feedback control at the hippocampus and hypothalamus through activation of GR protein expressed in these sites. Variability in rat HPA axis tone has been ascribed to changes in hippocampal GR expression, regulated by altered methylation of the rat GR exon 1–7 promoter [17]. This is located, as in the human, in the upstream CpG island. More recent studies have examined methylation status of a number of alternate human GR promoters in peripheral blood mononuclear cells [16]. These studies have revealed extensive, and variable methylation.

Hippocampal and hypothalamic GR plays a key role in the negative feedback control of the hypothalamic-pituitary-adrenal axis. Altered GR expression in the brain results in re-setting of the tone of this neuroendocrine axis and this is associated with altered methylation of nerve growth factor inducible-A (NGFI-A, or KROX, EGR1) binding site in the rat exon 1.7 promoter, homologous to the human exon 1F promoter [17]–[19]. This methylation controlled alteration in GR expression results in consequences for the whole organism, by regulating adrenal glucocorticoid production.

In this study we examined the pattern of GR promoter methylation in human SCLC cells, and showed that reversal of the methyl marks increased GR protein expression, and function. Moreover, we identified a negative correlation between GR 1C promoter methylation and GR protein expression across a panel of human SCLC cell lines, and human control cells.

## Materials and Methods

### Cell Culture and Maintenance

A549 human lung epithelial carcinoma cells, HEK-293 human embryonic kidney cells, HeLa human cervical carcinoma cells and U20S human osteosarcoma cells (European Collection of Cell Cultures, Wiltshire, UK) were all cultured in DMEM (Invitrogen) supplemented with 10% fetal calf serum (FCS) (Invitrogen).

Non-small cell lung cancer lines NCI-H358 and –H727 (European Collection of Cell Cultures, Wiltshire, UK) and NCI-H23, -H441, -H1299 (American Type Culture Collection, USA) were grown in RPMI 1640 medium (Invitrogen) supplemented with 10% FCS and 10 mM HEPES as recommended by supplier.

Small Cell Lung Cancer (SCLC) cell lines used in this study were the 10‘COR’ cell lines Cor L24, Cor L27, Cor L31, Cor L32, Cor L42, Cor L47, Cor L51, Cor L88, Cor L99 and Cor L103. Also used were DMS 79, DMS 153, HC12 and HX 148. All of these cell lines were derived from patients with pathologically confirmed SCLC [20], [21] with the exception of Cor L32, which was derived from a patient with poorly differentiated squamous carcinoma of the lung. This cell line did however exhibit the characteristics of SCLC [21]. All SCLC cell lines were cultured in RPMI 1640 medium (Invitrogen) supplemented with 10% FCS and 10 mM HEPES, as previously described [12].

Human peripheral blood mononuclear cells were obtained from healthy local donors, according to LREC approval 09/H1013/6.

Normal human bronchial epithelium was harvested from lung resection specimens after surgery for lung cancer, with full, informed patient consent.

### Treatments

Where appropriate, cells were treated with vehicle or 100 nM dexamethasone (Sigma-Aldrich) for 24 hours at 37°C, 5% C0_2_ before lysis. In certain experiments, cells were incubated with vehicle or 5 µM 5′Azadeoxycytidine (Sigma-Aldrich) for 72 hours or 5 days. After treating with 5′Azadeoxycytidine some cells were treated for a further 72 hours with 100 nM dexamethasone.

### Immunoblot analysis

Cells were lysed using 1× RIPA buffer (100 mM Tris base, 150 mM NaCl, 1% Igepal, 2.5% sodium deoxycholate, 1 mM EDTA).This buffer also contained protease inhibitors (Roche). Following centrifugation for 30 min at 10,000 *g* supernatants were harvested and assayed for protein content using a Bradford assay (Bio-Rad). Samples were then diluted in loading buffer [0.125 M TrisCl (pH 6.8), 0.1% sodium dodecyl sulphate (SDS), 20% glycerol, 0.2% β-mercaptoethanol, 0.001% bromophenolblue]. Cell extracts (50 µg) were then analysed by SDS-PAGE and western blotting as described [22], [23]. Primary antibodies used were the mouse monoclonal anti-hGR (Clone 41, 1∶2500), which binds in the N-terminal region of GR (BD Biosciences), rabbit polyclonal anti-hGR (P-20, 1∶500) raised against a peptide mapping at the C-terminus of GRα (Santa Cruz) and the mouse monoclonal α–tubulin (1∶5000) (Sigma-Aldrich). Secondary antibodies used were the horseradish peroxidase-conjugated anti-mouse (1∶5000) and anti-rabbit (1∶5000) (GE Healthcare). To quantify protein levels, densitometric analysis was carried out. Blots were scanned and the densitometry of each band was analysed using ImageJ software. The internal loading control (αTubulin) was also quantified and the results were normalised against these readings. This analysis was carried out on 3 separate blots for each experiment and the average calculated.

### Reporter Gene Assays

Cells were co-transfected with 2 µg of a TAT3-luciferase construct [13] and 0.5 µg of a pCMV-*Renilla* Luciferase construct (to correct for transfection efficiency) using Fugene 6 (Roche). In some cases, cells were also transfected with 1 µg of a GR expression vector (pFUNC1-GR-eYFP) [13]. Cells were treated with dex as described before luciferase assays were carried out using the Dual Luciferase Reporter Assay System (Promega), as previously described [8].

### Retroviral infections

pFUNC1-eYFP or pFUNC1-GR-eYFP were transfected into HEK293 for retroviral production using Fugene HD (Roche, UK), as a transfection reagent at a 3∶2 reagent∶DNA ratio. Infection of the retroviral particles into DMS 79 cells was performed as detailed before. Post infection, cells were cultured in normal growth media containing serum for 72 hours.

### Cleaved caspase-3 assay

DMS 79 cells expressing GR-eYFP or eYFP were applied to poly-L-lysine coated cover slips and fixed with 4% formaldehyde. Cells were permeabilized with PBS/0.2% Triton-×100. After washing, cells were blocked in PBS/0.1% Tween-20+5% donkey serum. Anti-ACTIVE Caspase-3 pAb (Promega, UK) diluted 1∶250 in blocking buffer was added to cells and incubated overnight. Secondary antibody incubation was performed in the dark using Alexa Fluor 546 donkey anti-rabbit IgG (Invitrogen, UK) diluted 1∶500 in PBS. Cover slips were mounted using ProLong Gold with DAPI (Invitrogen, UK).

Images were collected on an Olympus BX51 upright microscope using a 60×/1.40 UPlanApo objective and captured using a Coolsnap ES camera (Photometrics) through MetaVue Software (Molecular Devices). Specific band pass filter sets for DAPI, FITC and Texas red were used to prevent bleed through from one channel to the next. Images were processed and analysed using ImageJ (http://rsb.info.nih.gov/ij).

### Sodium Bisulphite Sequencing

Genomic DNA was extracted from several of the cell lines used in this study, some of which had been treated with 5′ Azadeoxycytidine (see results) using the DNeasy blood and tissue kit (Qiagen). Purified genomic DNA was then bisulphite converted using the Epitect Bisulphite Kit (Qiagen). This converted DNA was then used as a template in PCR reaction to amplify specific GR promoter regions. GR promoter regions amplified (and the primers used) were as follows:

Promoter

1C (forward- 5′-AGGTGGATCCGGAAGGAGGTAGYGAGAAAAGAAATT-3′; reverse- 5′- AGGTGAATTCACACRAACTCRCAAAATAAAAAAAA-3′).

Promoter

1D(forward- 5′-AGGTGGATCCTTTTATAAAAATTTTTTTGGTTGAGG-3′; reverse- 5′- AGGTGAATTCCCCCCTACTCTAACATCTTAAAAA).

Promoter

1E(forward-5′- AGGTGGATCCTTAGAGTTATAAAAATTATAATTTGTGT-3′; reverse- 5′- AGGTGAATTCATACAAACAACTTTAAAATACCAAC-3′).

All primers were supplied by MWG-Eurofins.

PCR was carried out using Immolase Polymerase (Bioline), following the manufacturer's instructions. Cycling conditions were as follows: 95°C for 10 minutes, 30 cycles, at 95°C for 30 seconds, at 56°C for 45 seconds, and at 72°C for 30 seconds, this was followed by a final extension of 72°C for 10 minutes.

PCR products were electrophoresed on a poly-acrylamide gel and purified using a gel extraction kit (Qiagen). Ligations were then carried out using these PCR fragments using the pGEM-T-easy vector system (Promega) following the manufacturer's instructions. Following transformation into JM-109 competent bacteria (Promega), colonies were picked and incubated overnight in LB broth at 37°C. Mini-preps (Qiagen) were carried out on these cell suspensions, followed by a restriction digest using EcoRI (Roche) to confirm the presence of the correct insert. Plasmids containing the insert were then sent for sequencing using an SP16 primer at the DNA Sequencing Facility, University of Manchester. An initial study was carried out to analyse potential methylation and the effects of 5′Azadeoxycytidine, using DNA from DMS 79 cells. In this case, one clone was analysed for each condition (each individual promoter, untreated or treated with 5′Azadeoxycytidine). A more in depth study was carried out to analyse methylation of the 1C promoter in a panel of SCLC cell lines. In this case, 3 clones were analyzed per cell line.

### Statistics

All statistical analysis was carried out using SPSS for Windows version 16. Further statistical analysis was carried out to compare methylation levels by Dr Steve Roberts, University of Manchester; using a linear regression model. The equation used for this analysis was as follows:
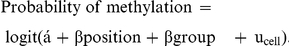



## Results

### Expression of GR is impaired in the human SCLC cell line, DMS79

**Figure 1:**
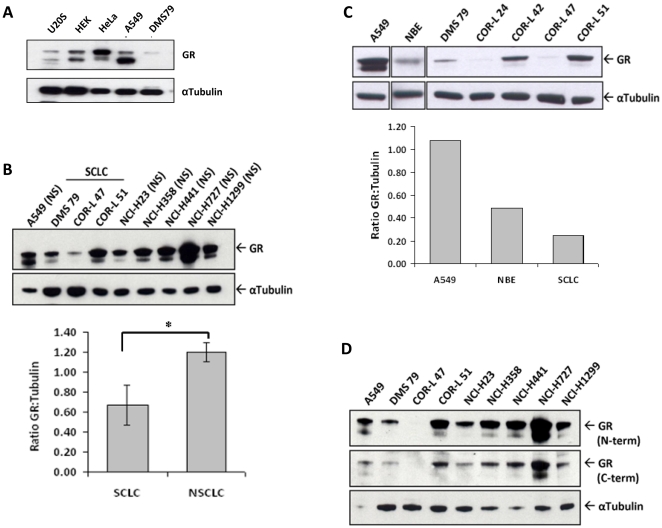
GR expression is decreased in the SCLC cells compared to non-SCLC cells and bronchial epithelial cells. (A) Western blot analysis of GR, and tubulin protein expression in U20S, HEK, HeLa, A549 and DMS 79 cells. Blot is representative of 3 separate experiments. (B) Comparison of GR protein expression between SCLC, and non-SCLC (NS) cell lines. Quantitation of GR expression relative to tubulin is presented as mean +/− S.E.M. (n = 3) with * indicating p<0.05, Student's t-test for independent samples. (C) Comparison of GR protein expression in normal human bronchial epithelium (NBE) compared to non-SCLC cell line (A549), and a panel of human SCLC cell lines. Quantitation of GR expression relative to tubulin is presented. Mean of n = 2. (D) Analysis of GR protein expression using a pan-GR antibody raised against the GR N terminal (N-term), and a GRalpha specific GR antibody raised against the C terminal (C-term).

GR protein was measured in the hSCLC cell line, DMS79 and compared with two Gc sensitive cells lines, HeLa and A549, and two Gc resistant cells lines, U2OS and HEK293. The SCLC cell line DMS79 expresses low levels of GR protein, similar to the U2OS cells, and clearly far less than the HeLa and A549 cells (Fig. 1a). Comparison with a panel of non-SCLC cell lines indicates that expression of GR is lower in the SCLC cells (Fig. 1b). GR protein was found to be expressed in normal human bronchial epithelium, harvested from lung tissue at resection of lung cancer (Fig. 1c).

GR migrated as two major species, and the relative abundance appeared to differ between cell types (Fig. 1a,b). This may reflect alternative splicing to the GRβ isoform, or alternative translation start site usage [22]–[24]. To further characterize the species, immunoblots were repeated using a C terminal specific antibody that specifically recognizes GRα (Fig. 1d). The similar banding pattern observed with the two antibodies indicates both proteins share a common C terminal domain, suggesting that the protein diversity lies with alternative translation start site usage.

### Restoration of GR expression induces apoptosis

**Figure 2:**
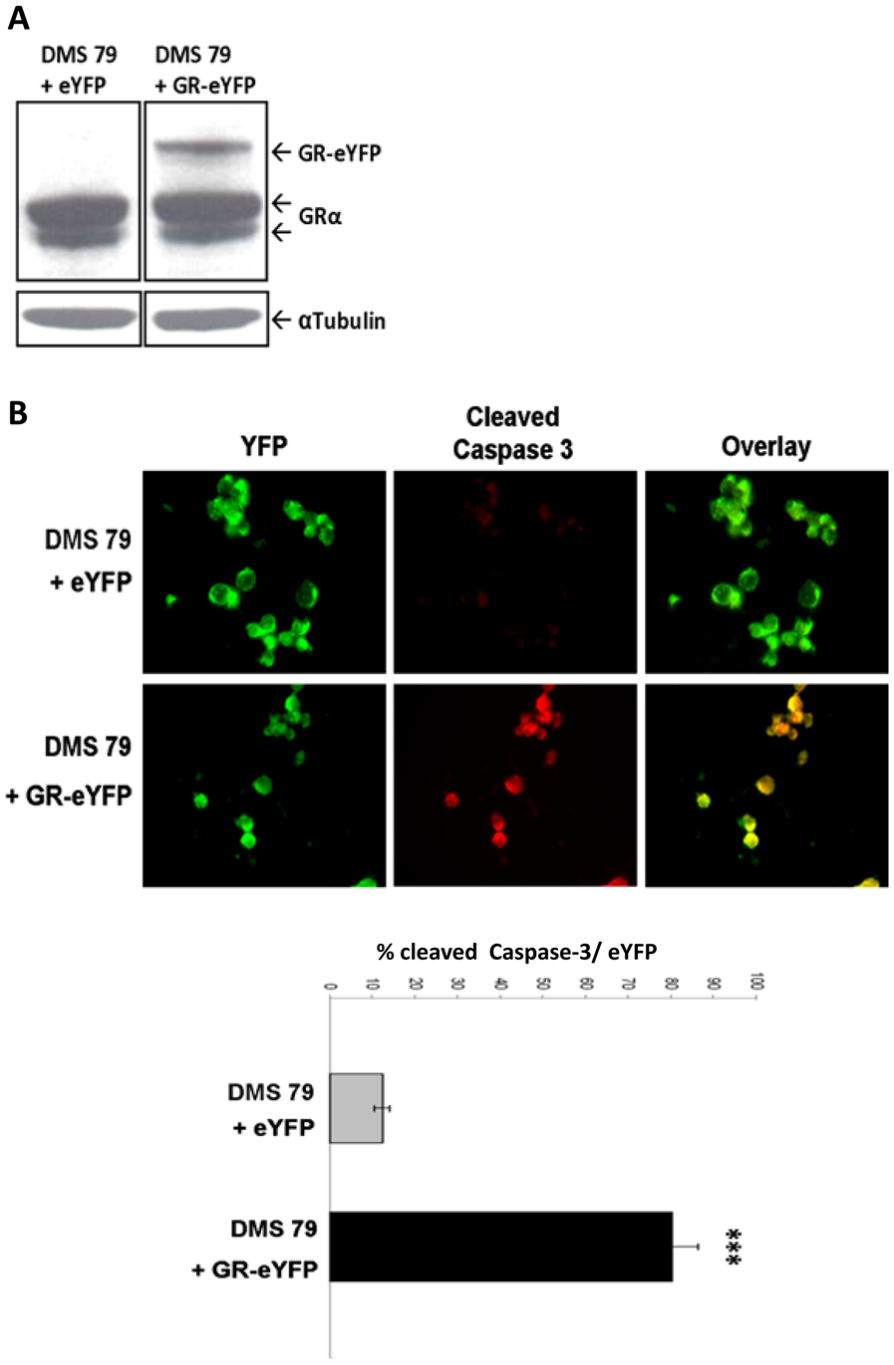
Expression of the GR transgene caused apoptosis. (A) DMS 79 cells were infected with either GR-eYFP or eYFP retroviruses and GR protein assessed at 72 h. (B) DM79 cells treated as above were fixed for immunohistochemistry to detect cleaved caspase-3. Cells were stained for activated caspase-3 and then analysed for co-localisation with either GR-eYFP or eYFP. Percentage of cells positive for cleaved caspase-3 and eYFP or GR-eYFP. Cell counts derived from 3 independent infections and represented as percentage of caspase-3 positive to eYFP positive as mean +/− S.E.M. (n = 3) with *** indicating p<0.001, Student's t-test for independent samples.

In DMS79 cells expression of a GR-eYFP transgene by retroviral infection resulted in increased GR-eYFP protein (Fig. 2a) and apoptosis as evidenced by increased cleaved caspase-3 in the SCLC cells expressing the GR-eYFP (Fig. 2b). In these studies only a minority of cells are effectively transduced, hence the low abundance of expressed fusion protein in the pool of cells. In addition, expression of the transgene induced apoptosis, further reducing GR-eYFP concentration in pooled cell lysates [13], [14].

### Methylation status of GR promoter in SCLC cells

**Figure 3:**
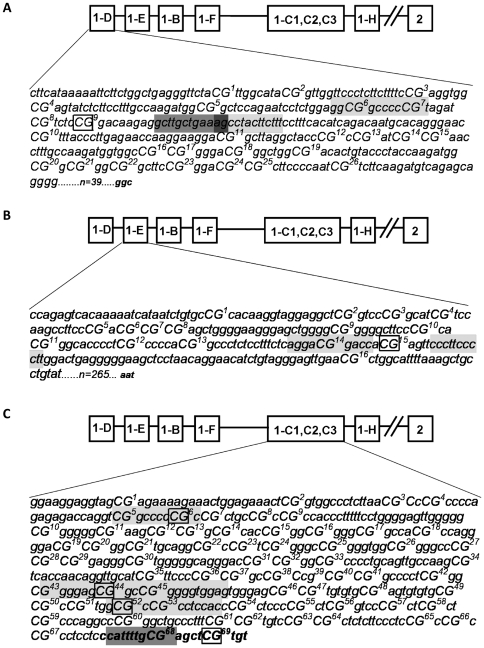
CpG methylation in the GR 1C, 1D, and 1E promoter regions in DMS 79 cells. Bisulphite sequencing was performed on extracted genomic DNA from DMS 79 cells incubated with 5′Azadeoxycytidine for 5 days (untreated as control). The original sequence (obtained from GENOME Browser) was compared to bisulfite treated DNA from the control and 5′Azadeoxycytidine treated samples. Bold, open boxes highlight specific methylated CpGs, which showed reversal of methylation after 5′Azadeoxycytidine incubation. The potential transcription factor binding sites in close proximity to the methylated sites are indicated by grey shading for Sp1 binding site and dark grey shading for C/EBPβ binding site. (A) CpG methylation in the GR 1D promoter region in DMS 79 cells. Individual CpGs are numbered and in capitals. (B) CpG methylation in a region of the GR promoter 1E from DMS 79 genomic DNA. Individual CpGs are numbered and in capitals. (C) Genomic DNA from a region of the GR promoter 1C from DMS 79 cells. Individual CpGs are numbered and in capitals. Italic text indicates the start of exon 1C.

The decreased GR expression in SCLC cells may result from alteration in GR promoter methylation, or from an indirect mechanism. Therefore methylation status of the GR promoters in DMS79 cells was examined by bisulfite sequencing. We did not detect any DNA methylation in the promoters 1B, and 1F. The 1D and 1E promoter each had only a single methyl CpG, marked with an open box (Fig. 3a,b). In contrast the 1C promoter had four methyl CpGs (Fig. 3c). The observed methylated CpGs are located in potential transcription factor binding sites, (CpGs 6, 44 and 52), or lie close to such a site (CpG 69) (Fig. 3c).

### SCLC cell lines have increased methylation of the GR 1C promoter

**Figure 4:**
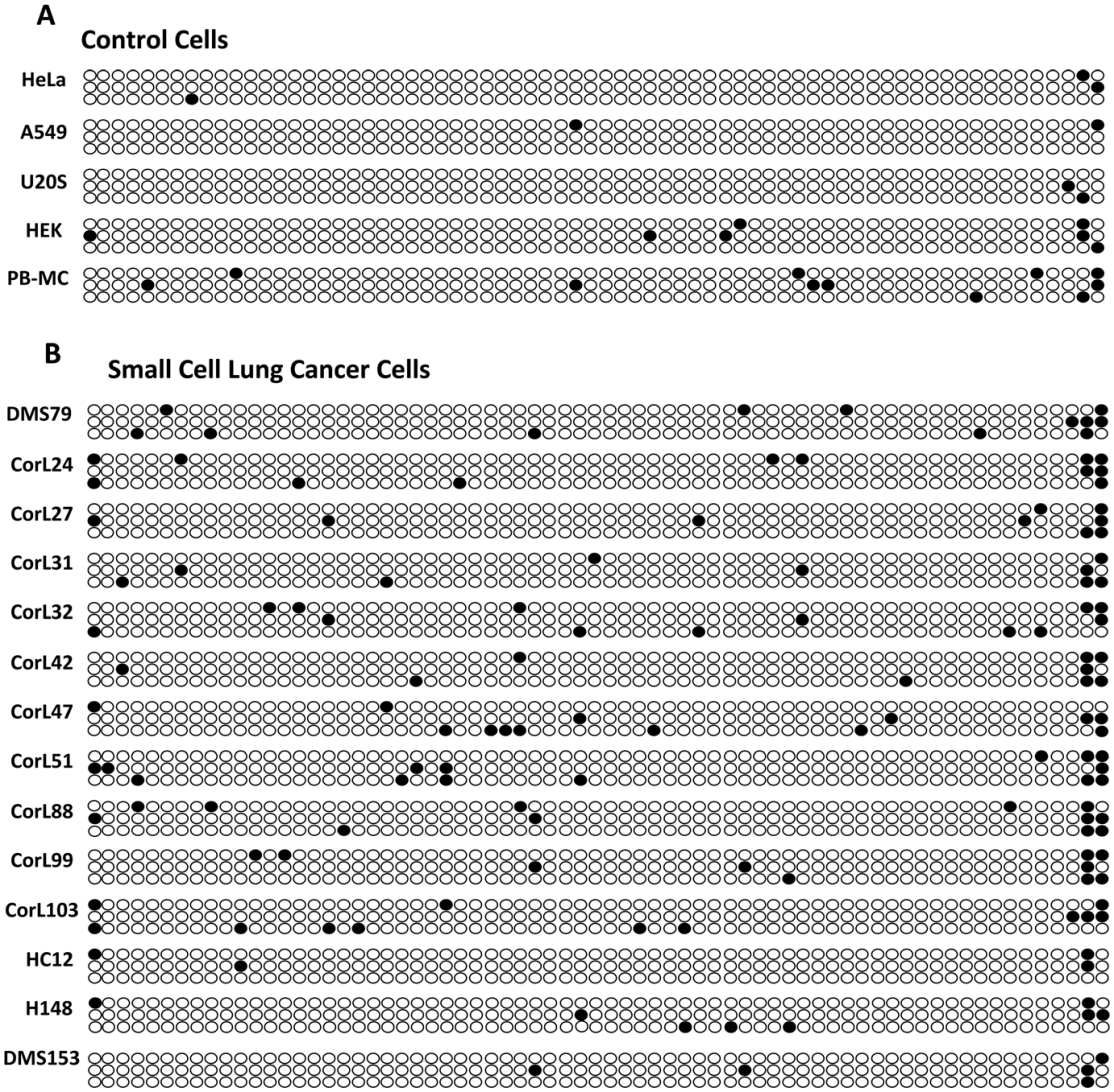
CpG methylation of GR 1C promoter in a panel of 14 SCLC Cell lines. Genomic DNA was extracted from (A) specific control cell lines, including primary peripheral blood mononuclear leucocytes from a healthy donor and (B) 14 hSCLC cell lines. After bisulfite conversion the 1C promoter region was PCR amplified. 3 clones per cell line were then sequenced. Methylation in the panel of SCLC cell lines and control cell lines is displayed as “beads”. Each individual bead represents a single CpG in the GR 1C promoter in numerical order from left to right. White beads indicate unmethylated CpGs whilst black beads indicate methylated CpGs. All 3 clones are displayed for each cell line analyzed.

To determine if the observed changes in GR promoter methylation were found in other human SCLC cell lines, a panel of 14 hSCLC cells lines with varying GR expression were compared with two GR expressing cell lines, (A549 and HeLa) a non-expressing cell line, (U2OS), and peripheral blood mononuclear cells as a primary cell comparison (Fig. 4a,b). The beads represent CpG dinucleotides from position 1 to position 69. It is readily apparent that there is frequent methylation at the last two beads on the right, which represent CpG 68 and 69. This is present across the panel of SCLC cells, and is also seen in the control cells. However, there is also a marked increase in methylation at positions 1–67 across the SCLC panel, without any notable clustering. In contrast there is very little CpG methylation seen at positions 1–67 in the control cell panel (Fig. 4a,b).

Logistic regression was used to compare methylation between the SCLC panel, and all the control cells (Table 1). This revealed that there was a significant difference between groups with methylation at CpG 69 individually; and with CpG 68 and 69 in combination, and across the whole promoter region. A significant difference was also observed between groups for all CpGs excluding 68 and 69 (Table 1).

**Table 1:** Analysis of differences in methylation between SCLC cell lines and all control cells.

	Group effect		Position effect	Cell line effect
Analysis	OR (95%CI)	P	P	σ
CpG 68	2.4 (0.8 to 7.0)	0.11	-	0.0001
CpG 69	4.5 (1.4 to 14.0)	0.010	-	0.001
CpG 68 and 69	3.3 (1.5 to 7.1)	0.003	0.58	0.0001
CpG <68	1.8 (1.1 to 3.1)	0.027	0.32	0.23
All CpGs	2.1 (1.3 to 3.4)	0.002	<0.001	0.22

A logistic regression analysis was carried out to compare methylation in the 14 SCLC cell lines (n = 48) to controls (HeLa, A549, U20S, HEK and PBMC's) (n = 21 (all 9 A549 clones included)). The group effect is displayed as an odds ratio (OR) with a 95% confidence interval (CI) for SCLC vs control, with p values <0.05 regarded as significant. It would be expected that the repeats from each cell line would be correlated. To handle this a cell-line specific random effect is included in the model. This effect is parameterised by a single standard deviation, σ. Where multiple sites are analysed the site is included as an additional covariate, as in a 2-way analysis of variance (position effect).

### GR promoter methylation is correlated with GR expression

**Figure 5:**
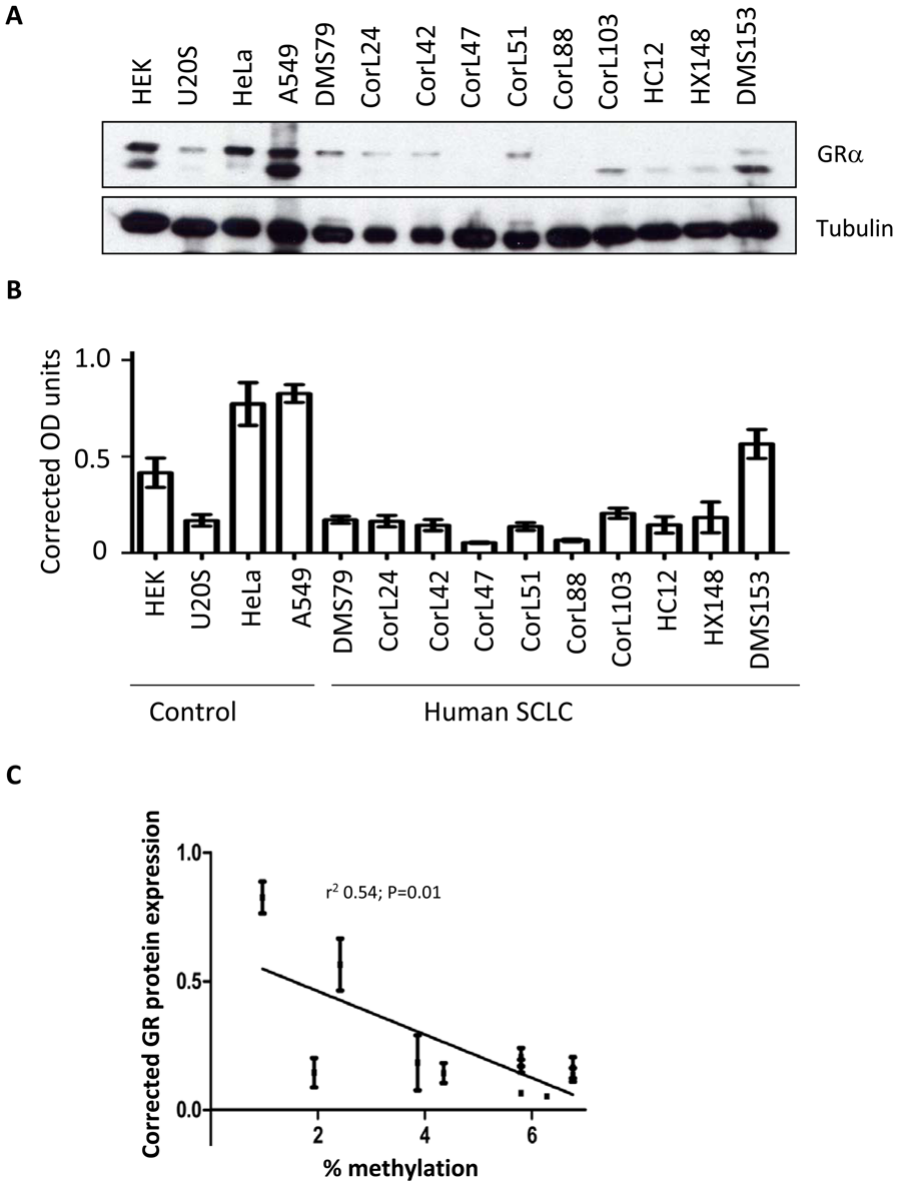
GR expression levels in a panel of hSCLC cell lines. (A) Representative western blot for GR and tubulin in 10 SCLC cell lines as well as control cell lines HEK (human embryonic kidney), U2OS (osteosarcoma), HeLa (cervical carcinoma) and A549 (non small cell lung carcinoma). (B) Densitometry was carried out on 5 separate blots to quantify the levels of GR expression relative to tubulin. Graph shows the average, corrected GR expression ± S.E.M. (C) Relationship between methylation of GR Promoter 1C and GR expression in a panel of human lung cancer cell lines. Overall methylation (percentage) across promoter 1C was plotted against GR expression, with standard error, for each cell line. The regression line was significantly different to zero (P = 0.01), and the goodness of fit was r^2^ = 0.54.

Across all the hSCLC cell lines there was a wide range of GR expression, although in all cases GR was less abundant than in the HeLa and A549 control cell lines (Fig. 5a, b). There was also a marked variation in the abundance of the two GR protein species across the cell lines examined, but for this analysis both GR protein isoforms were considered together. The relationship between GR 1C promoter methylation and GR protein expression was examined across the whole panel of hSCLC cell lines. There was a significant correlation between number of methylated CpGs, and the GR protein expression within the panel of hSCLC cell lines; r^2^ = 0.54, and p = 0.01 (Fig. 5c).

### Restoration of GR expression by reversal of DNA methylation

**Figure 6:**
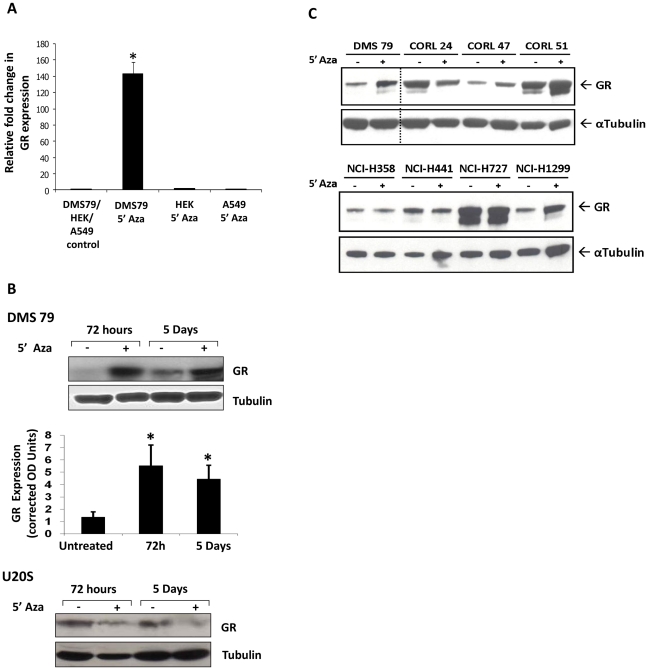
Treatment with DNA methyltransferase 1 inhibitor, caused an increase in GR expression in DMS 79 cells. (A) GR mRNA expression normalized to GAPDH in response to 72 hours treatment with 5′azadeoxycytidine (5′Aza) in DMS 79 cells, GR deficient HEK cells, and GR expressing A549 cells. (B) Two GR deficient cell lines, DMS 79 and U2OS, were treated with 5′Azadeoxycytidine (5′Aza) for 72 hours and 5 days (untreated cells as control). Lysates were then analysed by western blot for GR expression. For DMS 79 cells, the GR expression was normalised against α tubulin, and plotted as average GR expression plus S.E.M. (n = 3). * indicates p<0.01 compared to vehicle treated control. (C) A panel of human SCLC cell lines (upper panel) was compared to a panel of non-SCLC cell lines (lower panel) for the response of GR protein to incubation with 5′Azadeoxycytidine (5′Aza).

If the loss of GR expression in DMS79 cells resulted from DNA methylation, perhaps consequent to carcinogenesis, GR expression would be predicted to increase following reversal of methylation. After 72 hours of treatment with 5′Azadeoxycytidine, GR mRNA levels increased dramatically in DMS79 cells when compared to HEK and A549 cells where no changes in expression were seen (Fig. 6a). Furthermore, after 72 hours, or after 5 days incubation with 5′Azadeoxycytidine, there was a striking increase in GR protein (Fig. 6b). In contrast to the increase in GR expression seen in DMS79, there was decreased expression in U2OS cells, possibly due to toxicity (Fig. 6b).

The effect of 5′Azadeoxycytidine was then compared in human SCLC cell lines and non-SCLC cell lines to determine if the regulation of GR expression by methylation was widespread in lung cancer. Three of the four SCLC cell lines showed augmented GR expression after 5′Azadeoxycytidine treatment, in comparison to only one of the four non-SCLC cell lines (Fig. 6c).

**Figure 7:**
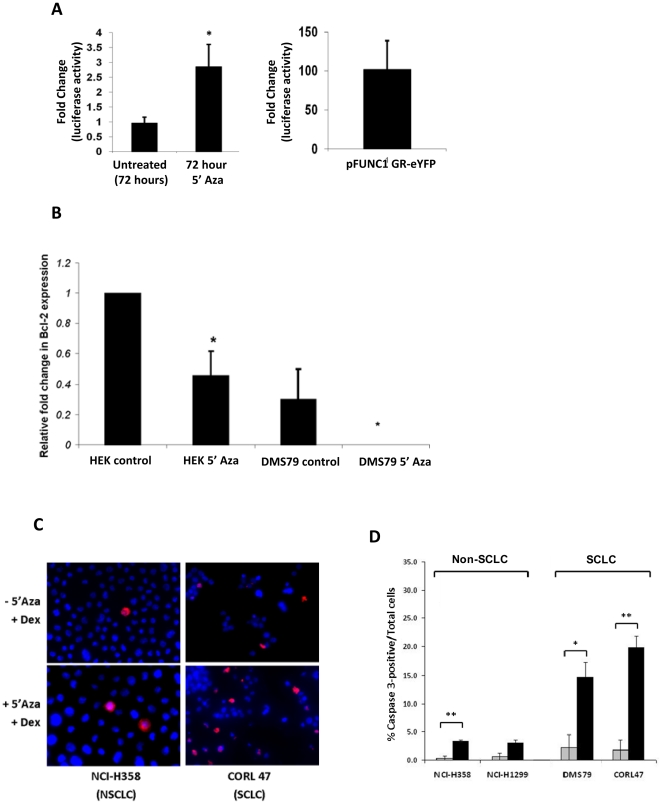
Treatment with 5′Azadeoxycytidine restores Gc sensitivity, suppresses BCL2 expression and increases active caspase-3. (A) Treatment of DMS 79 cells with 5′Azadeoxycytidine (5′Aza) restores Gc sensitivity. DMS 79 cells treated with 5′Aza for 72 hours were transfected with the TAT3-Luciferase reporter construct. Cells were then incubated overnight with or without 100 nM dexamethasone. Graph shows the average fold change (dex treated/untreated), (relative light units), (triplicate) plus the S.E.M. (* = p<0.05). DMS 79 cells were also co-transfected with pFUNC1 GR-eYFP (as a positive control) and the TAT3-luciferase reporter construct. Cells were then incubated overnight with or without 100 nM dexamethasone. Graph shows the average fold change (dex treated/untreated) plus the S.E.M. All results were normalised against a renilla control. Results are representative of 3 separate experiments. (B) Bcl-2 mRNA expression normalised to GAPDH in response to 72 hours treatment with 5′Azadeoxycytidine (5′ Aza) in DMS79 and HEK cells. (C) Treatment of cells with Aza and then dexamethasone results in apoptosis. Representative image of SCLC cells (CORL47) and non-SCLC cells (NCI-H358) treated with Aza as described above. Cells were then treated with dexamethasone for 72 h, fixed and stained for active caspase-3. (D) Demethylation leads to marked increase in Gc mediated apoptosis in SCLC cells compared to non-SCLC cells. Apoptosis measured by caspase-3 activation after 5′Aza and dexamethasone as described above. Grey bars represent cells treated with dexamethasone, black bars for cells treated with 5′Aza and then with dexamethasone. Each cell line was incubated in triplicate and 100 cells counted to assess % positive staining. Independent student's t-test * = p<0.05, ** = p<0.01, *** = p<0.001.

The increase in GR expression seen in DMS79 cells is predicted to restore Gc sensitivity to these cells. Indeed, there was increased Gc transactivation of a simple reporter gene (Fig. 7a). The magnitude of Gc induction was considerably less than that seen with GR overexpression, but the latter does result in supra-physiological GR concentrations in target cells (Fig. 7a).

The increased GR expression in response to treatment with 5′Azadeoxycytidine resulted in suppression of the pro-survival Bcl-2 gene in both HEK, and DMS79 cells (Fig. 7b). There was also increased cleaved caspase-3 activation in the SCLC cells but not the non-SCLC cells (Fig. 7c & 7d). It is important to note that after 5 days incubation with 5′Azadeoxycytidine there was induction of cell death, possibly due to reacquisition of GR expression, or regulation of other survival genes.

## Discussion

Epigenetic disorders are implicated in many human diseases including cancer. Cigarette smoke is the principal environmental agent promoting SCLC and recently, a cigarette smoke carcinogen, NNK, has been shown to exert a specific effect promoting DNMT1 expression, and activity [4]. Therefore this carcinogen may act by inducing epigenetic changes in key genes implicated in the pathogenesis of the cancer.

We have previously shown that the glucocorticoid receptor is effectively a tumor suppressor gene for SCLC, whose expression is inhibited [12]. We have extended the analysis in this study with further evidence that the GR expression is lower in a panel of SCLC cells compared to non-SCLC cells or normal bronchial epithelial cells. Reacquisition of GR expression, and glucocorticoid sensitivity in SCLC cells *in vitro*, or in xenograft tumors results in apoptosis [13], [14]. GR is expressed in most cells and tissues, and exerts pleiotropic effects on cells. However, relatively little is known of how GR gene expression is regulated, as it has an unusually complex promoter structure. More recent studies in primary peripheral blood mononuclear cells defined highly individual patterns of GR promoter methylation [16]. This work also mapped potential, and proven transcription factor binding sites within the promoters, the majority of which contained CpG sites which could be methylated. This suggested that differential methylation of the human GR promoter is a mechanism to control promoter utilization, and so GR gene expression.

Three GR promoters were examined in the index cell line, DMS79, and all three contained methylated CpG sites. Further analysis was performed on the 1C promoter as it has been shown to be ubiquitously expressed in all tissues tested, including lung [15]. There was an obvious and highly significant increase in CpG methylation across a panel of hSCLC cell lines compared to the non-SCLC cell line A549. Importantly there was no increase in the two non-expressing control cell lines (U2OS and HEK293), indicating that 1C promoter methylation is not required to limit GR expression, but that other mechanisms may apply to regulate GR expression in different cell types. A broader comparison between hSCLC cells and a heterogeneous panel of GR expressing, and non-expressing cell lines also found a highly significant increase in methylated CpG sites in the hSCLC cells. It was striking that methylation of CpG68 and 69 was far higher across all the cell lines examined than positions <68. CpG68 lies within a consensus SP-1 transcription factor binding element, and CpG69 lies immediately 3′ to it. There was no other positional clustering of the methylated CpG sites, and statistical analysis did not identify any significant positional effect. Some of the methylated CpG sites at <68 overlie consensus transcription factor binding sites (CpG6, 44 and 52), but CpGs1,2 and 3 did not.

Within the panel of hSCLC cells there was variation in GR protein expression, which was quantitated by immunoblot under non-ligand treated conditions. If GR promoter 1C methylation played a role in regulating GR expression a correlation would be expected between degree of methylation, and protein expression. Indeed, a significant negative correlation was seen.

Studies were aimed at discovering if DNA methylation could regulate GR expression in hSCLC cells, in a similar manner to that defined in the rat brain. Treatment with 5′Azadeoxycytidine resulted in a striking induction in GR mRNA and protein in SCLC cells, although, as expected, there was heterogeneity amongst the cell lines profiled, with one cell line (COR L24) showing no induction. Two confounding issues in these studies are that use of 5′azadeoxycytidine exerts a toxic effect on the SCLC cells, likely as a consequence of regulating multiple tumour suppressor genes and also that increased GR protein expression results in increased cell death in SCLC cells [13], [14]. In contrast, augmented GR expression was only seen in one of four non-SCLC cell lines. Interestingly, in another GR deficient cell line, U2OS, there was no increase in GR expression, suggesting that loss of GR expression by promoter methylation may be specific to SCLC. Analysis of GR mRNA expression showed no induction in two further cell lines, suggesting a cell-type specific effect. The removal of methyl marks with 5′Azadeoxycytidine resulted in restoration of glucocorticoid regulation of a simple reporter gene, confirming expression of a functional receptor and increased glucocorticoid effects. The increase in GR expression was accompanied by reacquisition of inhibition of Bcl-2, a key component of the anti-apoptotic pathway and an increase in cleaved caspase-3, indicative of apoptosis. This suggests that removal of methyl marks from the GR results in apoptosis although changes in other tumor suppressor genes are also likely to play a role in this effect.

Taken together our data support a role for DNA methylation in regulating GR gene expression in human SCLC cells. This mechanism is proposed to explain the reduction in GR expression observed, and the consequent loss of Gc sensitivity. There is a clear survival advantage for the cancer cells from evading GR integrity, as restoring GR expression induces apoptosis. Indeed in our study, treatment with 5′Azadeoxycytidine itself resulted in reacquisition of GR expression, which was followed by specific SCLC cell death. It seems possible that exposure to the tobacco smoke carcinogen NNK may play a role in silencing the GR gene by increasing DNMT1 mediated methylation of the GR promoters lying within the 5′CpG island and thus reducing the role of GR in regulating apoptosis in the SCLC cells.
