# Correction: Loss of Glucocorticoid Receptor Expression by DNA Methylation Prevents Glucocorticoid Induced Apoptosis in Human Small Cell Lung Cancer Cells

**DOI:** 10.1371/annotation/4aad3313-abac-404f-85b0-3b95aec89be4

**Published:** 2013-08-23

**Authors:** Paul Kay, George Schlossmacher, Laura Matthews, Paula Sommer, Dave Singh, Anne White, David Ray

A funding organization for the fourth author was incorrectly omitted from the Funding Statement. The following sentence should be included in the Funding Statement: "PS was funded by CANSA." 

